# Construction of Stable Fluorescent Reporter Plasmids for Use in *Staphylococcus aureus*

**DOI:** 10.3389/fmicb.2017.02491

**Published:** 2017-12-14

**Authors:** Michelle D. Rodriguez, Zubin Paul, Charles E. Wood, Kelly C. Rice, Eric W. Triplett

**Affiliations:** ^1^Department of Microbiology and Cell Science, Institute of Food and Agricultural Sciences, University of Florida, Gainesville, FL, United States; ^2^Department of Physiology and Functional Genomics, College of Medicine, University of Florida, Gainesville, FL, United States

**Keywords:** *: Staphylococcus aureus*, fluorescence, plasmid, stable, inoculation

## Abstract

Here, the genes encoding three different fluorescent proteins were cloned into the stably maintained *Staphylococcus aureus* shuttle vector pKK30. The resulting plasmids were transformed into two *S. aureus* strains; SH1000 and RN4220. Stability assays illustrated that the three recombinant plasmids retained near 100% maintenance *in vitro* for 160 generations. *S. aureus* strain SH1000 expressing green fluorescent protein was then inoculated in an ovine model and *in vivo* stability for 6 days was demonstrated. In essence, these reporter plasmids represent a useful set of tools for dynamic imaging studies in *S. aureus*. These three reporter plasmids are available through BEI Resources.

## Introduction

Instability of recombinant plasmids has posed a major burden in studies on staphylococci, especially with regard to *in vivo* and immune cell interaction experiments where antibiotic treatment is difficult. The feeding of animals with antibiotics to maintain plasmid stability may not be sufficient to confer plasmid stability in all infection sites. The use of antibiotics may also lead to unwanted perturbations in the host microbiota, leading to confounding results in microbiome studies (Kamruzzaman et al., 2017). In addition, certain classes of antibiotics cannot effectively cross host membranes and are ineffective in eukaryotic systems. Designing plasmids that persist both in *in vivo* and *in vitvo* conditions remains a challenge in *Staphylococcus aureus* research (Krute et al., 2016). Fortunately, plasmids based on the LAC-p01 shuttle vector, pKK30 and pKK22, that are stable in the absence of selection pressure are available (Krute et al., 2016), thereby eliminating the need for antibiotic use during the course of *in vivo* and *in vitro* experiments. Here, the construction of fluorescent reporter plasmids are described using pKK30 as a backbone because it lacks the four predicted open reading frames for use in non-USA300 *S. aureus* isolates (Krute et al., 2016). Three fluorescent reporter gene inserts were ligated into pKK30 including superfolder *gfp*, dsRed, and FP650 (far red), the latter two of which were previously codon-optimized for *S. aureus* (Bose et al., 2013). The fluorescent protein genes were cloned downstream of the sarAP1 promoter – dfrA gene fusion and then electroporated into *S. aureus* RN4220 followed by *S. aureus* RN4220, plasmid extracted and electroportated into SH1000.

## Materials and Methods

### Bacterial Strains and Media

Plasmids pGFP-F, pRFP-F, and pFP650-F were obtained from BEI Resources [Nebraska Transposon Mutant Library (NTML) Genetic Toolbox, NR-49947]. *S. aureus* strains containing pRFP, pGFP, and pFP650 were cultured in tryptic soy agar supplemented with 5 ug/mL chloramphenicol for selection. For cloning purposes, *Escherichia coli* DH5a (for pCR-Blunt-based cloning) and DH5αλpir (for propogation of pKK30 and derivatives) were cultured in Luria-Bertani broth supplemented with 50 μg/ml kanamycin or 10 μg/mL trimethoprim, as appropriate. *S. aureus* cells were made competent for transformation as described previously (Bose, 2014). The bacterial strains used in this study are listed in **Table 1**.

**Table 1:** Bacterial strains and plasmids used in this study.

Strain or plasmid	Description	Source
**Bacterial strains**		
*Escherichia coli* DH5α	Strain used for cloning purposes	Silhavy et al., 1984
*E. coli* DH5αλpir	Strain used for pKK30 cloning and maintenance	Krute et al., 2016
*E. coli* DH5αλpir (pSGFPS1)	GFP-labeled DH5αλpir	This study
*E. coli* DH5αλpir (pSRFPS1)	RFP-labeled DH5αλpir	This study
*E. coli* DH5α λpir (pSFRFPS1)	FRFP-labeled DH5αλpir	This study
*S. aureus* RN4220	Highly mutagenized, transformable *S. aureus*	Kreiswirth et al., 1983
*S. aureus* SH1000	Wild-type *S. aureus* derived from 8325-4 lineage	Horsburgh et al., 2002
*S. aureus* RN4220 (pSGFPS1)	GFP-labeled RN4220	This study
*S. aureus* RN4220 (pSRFPS1)	RFP-labeled RN4220	This study
*S. aureus* RN4220 (pSFRFPS1)	FRFP-labeled RN4220	This study
*S. aureus* SH1000 (pSGFPS1)	GFP-labeled SH1000	This study
*S. aureus* SH1000 (pSRFPS1)	RFP-labeled SH1000	This study
*S. aureus* SH1000 (pSFRFPS1)	FRFP-labeled SH1000	This study


### Plasmid Purification and PCR

Plasmids were purified from *S. aureus* and *E. coli* cultures using a Qiagen Plasmid Mini Kit (Qiagen, Inc., Valencia, CA, United States). *S. aureus* cell suspensions were pretreated with 1 μL of 2 mg/mL lysostaphin (Sigma–Aldrich) for 30 min at 37°C. DNA was quantified using a Nanodrop spectrophotometer (Thermo Scientific, Wilmington, DE, United States), and fragment sizes were assessed on a 1% agarose gel. All PCR reactions were performed with an Applied Biosystems GeneAmp PCR System 9700 (Life Technologies, Corp., Carlsbald, CA, United States) for appropriate primer sets on each template (Bose et al., 2013) using proofreading polymerase Accuprime Pfx (Invitrogen). PCR reactions were prepared with 50 μL 100x Accuprime mix, 1 μL of 10 μM primer stock, 20 ng of plasmid DNA adjusted to 50 μL total volume with sterile, nuclease-free water. Cycles consisted of a 2 min denaturation step at 95°C followed by 30 cycles of 15 s at 95°C for further denaturation, 30 s at 60°C for annealing, 1 min at 72°C extension and ended with a 10 min extension at 72°C. PCR products were purified using PCR Purification kit (Qiagen, Hilden, Germany), quantified fluorometrically by QuBit dsDNA High Sensitivity (Invitrogen, Life Technologies, Inc., Carlsbad, CA, United States), and fragments size was assessed on an 1% agarose gel. The DsRed and eqFP650 reporter genes used were codon-optimized for *S. aureus* and synthesized by Invitrogen. Synthesized genes were amplified by PCR from pTnT plasmids using the primer sets JBTN18/JBTN19, JBTN20/JBTN21 for DsRed, and eqFP650 as previously described (Bose et al., 2013). The gene encoding superfolder green fluorescent protein was amplified from pJB68 using the primer pair JBTN37/JBTN38, respectively. All fluorescent gene primers included a non-homologous *Asc*I sequence (GGCGCGCC), to allow cloning of fluorescent genes downstream of the *dfrA* cassette (**Table 2**).

**Table 2:** Oligonucleotides used in this study.

Oligonucleotide	Sequence (5′–3′)^∗^	Source
JBTN18	ggcgcgccTGATTAACTTTATAAGGAGGAAAAACATATGGA	This study
JBTN19	ggcgcgccTTATAAAAACAAATGATGACGACCTTCTGTAC	This study
JBTN20	ggcgcgccTGATTAACTTTATAAGGAGGAAAAACATATGGG	This study
JBTN21	ggcgcgccTTAACTATGACCTAATTTTGATGGTAAATC	This study
JBTN37	ggcgcgccTGATTAACTTTATAAGGAGGAAAAACATATGAG	This study
JBTN38	ggcgcgccTTATTTGTAGAGCTCATCCATGCCATGTG	This study
dfrA-pKK30	TTGTCGCTCACGATAAACAAA	Thisstudy


^∗^*Lowercase lettering denotes AscI site to promote cloning into AscI insertion site on pKK30*.

### Construction of Reporter Plasmids into *S. aureus* RN4220 and SH1000

The pKK30 plasmid described previously (Krute et al., 2016) was used as the backbone for these new plasmids. In addition, pKK30 includes the selectable marker, *dfrA*, which is under the control of the constitutive promoter (sarAP1). This marker confers trimethoprim resistance in these *S. aureus* strains. Furthermore, the *blaZ* transcriptional terminator in pKK30 prevents transcription beyond the reporter gene inserts, allowing for enhanced plasmid stability and maintenance.

Blunt ends of GFP, DsRED, and FP650 were generated for amplified fluorescent reporter genes and ligated into pCR-Blunt vector using the Zero Blunt TOPO PCR Cloning Kit (Invitrogen). Approximately, 5 μL of each ligation reaction was transformed into *E. coli* DH5a by heat shock and plated on LB plates supplemented with 50 μg/mL kanamycin for selection. Resulting colonies were then screened for the presence of each fluorescent gene insert by PCR using the primers described above. Subsequently, pCR-Blunt containing fluorescent gene DNA was digested with AscI, the appropriately sized restriction fragments were gel purified, ligated, and dephosphorylated, AscI-digested pKK30. By amplification with reporter gene using the primers described above as well as the *dfrA* primer from pKK30, 5′-TTGTCGCTCACGATAAACAAA-3′. The ligation products were transformed into *E. coli* DH5αλpir and then screened for the correct presence and orientation of reporter gene inserts by PCR using a forward primer specific for the *dfrA* gene (5′-TTGTCGCTCACGATAAACAAA-3′) and the appropriate reverse primer for each reporter gene. Proper orientation of inserts was also confirmed by Sanger sequencing downstream from the *dfrA* insertion site. The *E. coli* strains containing each plasmid were then stored in -80°C in 50% glycerol. The three fluorescent inserts were used to construct pSGFPS1, pSRFPS1, and pSFRFPS1. The nucleotide sequences of pSFRFPS, pSGFPS1, pSRFPS1, and can be obtained from GenBank accession numbers MF769789, MF769790, and MF769791, respectively. Collectively, these plasmids are referred to as pSxFPS1.

### Electroporation of Reporter Plasmids into *S. aureus* SH1000 and RN4220

Electrocompetent cells were prepared as described previously (Bose, 2014). Approximately, 1 μg of pSxFPS1 DNA derived from *E. coli* DH5a λpir was mixed with *S. aureus* electrocompetent cells and pulsed at 2.3 kV, 100 Ω, and 25 μF using the Gene Pulser Xcell Electroporation System (Bio-Rad Laboratories). Next, B2 broth (Bose, 2014) was added and cuvettes were incubated for 2 h at 37°C. Cell suspensions were then spread on solid TSA with 10 μg/mL trimethoprim, and incubated overnight at 37°C. Transformation efficiencies were determined for all three pSxFPS1 plasmids. For the RN4220 strains, the transformation efficiencies were 1.075 × 10^3^, 5.34 × 10^5^, and 4.88 × 10^5^ per ng/DNA for pSGFPS1, pSRFPS1, pSFRFPS, respectively. For the SH1000 strains, plasmids were first extracted from RN4220 and these were used to transform SH1000 with the transformation efficiencies of 6.45 × 10^2^, 5.37 × 10^5^, and 3.9 × 10^5^ per ng/DNA for pSGFPS1, pSRFPS1, pSFRFPS, respectively. Proper orientation of the inserts was firmed by PCR and sequencing. The presence of the reporter genes was confirmed by fluorescence microscopy. Transformants were confirmed by assessing proper orientation with isolate sequencing as described above and the presence of reporter gene inserts were observed visually by fluorescence microscopy (EVOS FL Cell Imaging System, ThermoFisher). RFP was excited at 531 nm and emission detected at 593 nm. GFP was excited at 470 nm and emission detected at 510 nm. FP650 was excited at 592 nm and emission detected at 650 nm.

### *In Vitro* Stability Assays on pSxFPS1 Reporter Plasmids

Long-term stability assays were performed, as described by Bose (2014), on the three pSxFPS1 reporter plasmids, and pKK30 as a control, to determine maintenance of plasmid during non-continuous exponential phase in the absence of trimethoprim in *S. aureus* strains RN4220 and SH1000. Recombinant SH1000 and RN4220 strains were inoculated in 10 mL TSB supplemented with 10 μg/ml trimethoprim, and grown overnight at 37°C with shaking (250 rpm). Next, cultures were diluted to 10^-4^ in 10 mL TSB without trimethoprim in 125 mL flasks and cultured at 37°C at 250 rpm up to an OD_600_ of 1.5. Cultures were then serially diluted to 10^-7^ in 10 mL TSB, 100 μL aliquots were spread plated onto TSA without antibiotic, and incubated overnight. Resulting colonies were then patched on to antibiotic plates to determine the percentage of colonies carrying pSxFPS1 or pKK30 (**Table 3**). These steps were continued for four additional days with the last step being plating and patching on to media with trimethoprim. The number of generations was calculated using OD_600_ readings and dilution factor, resulting in approximately 160 generations observed for all eight recombinant strains including those with pKK30. In a parallel experiment to assess the effect of each plasmid on growth of the host bacterium, overnight cultures of RN4220 and SH1000 (containing one of the reporter plasmids or pKK30) were diluted to an OD_600_ of 0.1 in 200 μL of tryptic soy broth in triplicate on 96-well plates alongside positive and negative controls. For continued bacterial culture growth, plates were incubated in a spectrophotometer at 250 rpm for 12 h to assess exponential phase growth in the absence of trimethoprim. This experiment was repeated three times and the mean of the three data points for each growth point were used for the analysis (**Figure 2**).

**Table 3:** *In vitro* stability of pkk30 reporter plasmids.

Plasmid	Strain	Trial	Generations	Plasmid present^∗^	% with plasmid
					
pSRFPS	*S. aureus* SH1000	1	160	145/150	97
		2	160	140/150	93
		3	160	150/150	100
					
pSGFPS	*S. aureus* SH1000	1	160	150/150	100
		2	160	150/150	100
		3	160	150/150	100
					
pSFRFPS	*S. aureus* SH1000	1	160	150/150	100
		2	160	150/150	100
		3	160	148/150	99
					
pSRFPS	*S. aureus* RN4220	1	160	150/150	100
		2	160	150/150	100
		3	160	150/150	100
					
pSGFPS	*S. aureus* RN4220	1	160	150/150	100
		2	160	149/150	99
		3	160	146/150	97
					
pSFRFPS	*S. aureus* RN4220	1	160	150/150	100
		2	160	148/150	99
		3	160	150/150	100


^∗^*Screened 150 colonies per plated culture*.

### Inoculation of an Ovine Model with Fluorescent *S. aureus* SH1000

An ovine model was used for this work because our long-term objective is to understand the role of bacteria, if any, in premature birth. Because of its size, sheep are considered an excellent model for premature birth. All procedures were approved by the University of Florida Animal Care and Use Committee and performed in accordance with the Guiding Principles for Use of Animals of the American Physiological Society. This work was approved by the University of Florida’s Institutional Animal Care and Use Committee (IACUC) as part of study number 201508915. Pregnant ewes of known gestational age and mixed breeds were transported to the University of Florida and acclimatized to the housing, light–dark cycle, temperature, and diet of the laboratory environment. Ewes were fed a diet of pelleted feed according to NRC standards for the ewe’s body weight and gestation. On days 130–132 of gestation, all ewes were inoculated intravenously with *S. aureus* SH1000 (pSGFPS1). The intravenous dose was 10^4^ cfu/mL (*n* = 4). Four to six days after inoculation, the ewes were humanely sacrificed and tissues were recovered from mother, placenta, and fetus for analysis of GFP and for isolation and culture of *S. aureus* SH1000. Antibodies against GFP with DAB were used to visualize bacteria expressing fluorescent proteins in fetal and maternal tissues.

### Immunohistochemistry

Tissue samples were fixed using 4% paraformaldehyde overnight after necropsy and set in 70% alcohol for storage. Tissue slices were cut to 2–3 mm slices and dehydrated in 90–100% Reagent Alcohol overnight. Slices were then cleared in xylene for 2–3 h. Following xylene clearance, tissues were placed in liquid paraffin and set in the vacuum oven at 60°C for 1 h with the vacuum on and 1 h with the vacuum off. Tissue slices were then mounted in paraffin blocks and left to dry overnight. Sections were cut at 5–7 micron thickness and mounted on slides.

Tissue sections were hydrated in a graduated alcohol dilution, quenched with BLOXALL Blocking Solution for 10 min, and blocked in 2.5% horse serum (R.T.U. Vectastain Kit, PK7200) for 30 min. Then, slides were incubated for 1 h with a 1 mg/mL anti-GFP antibody (Millipore AB3080) at a 1:500 dilution. Tissues were incubated for 30 min with a universal biotinylated antibody from the RTU Vectastain Kit. DAB (HRP) substrate (Vector SK-4100) was used for visualization of the anti-GFP antibody, and slides were counter stained with methyl green. Images were taken at 10 and 40x zoom using Olympus Digital Fluorescence microscope (Olympus Life Science).

### Culturing of Fluorescent *S. aureus* SH1000 from Ovine Tissues

Following a 4–6 day incubation period, 100 mg of maternal liver, lung, cerebral cortex, and placenta samples were mechanically homogenized in 500 μL of Brain Heart Infusion Broth (Sigma-Aldrich) for 30 s. Homogenates were serially diluted 10-fold, 100 μL aliquots were spread-plated onto solid BHI medium, and incubated overnight at 37°C. To assess abundance of SH1000 containing the three reporter plasmids, colonies on 10^4^ cfu/mL plates were enumerated, with resulting values of 1.9 x 10^7^ cfu/mL for pSGFPS1, 1.1 × 10^7^ cfu/mL for pSRFPS1, and 1.3 × 10^7^ cfu/mL for pSFRFPS1. Colonies were also screened by fluorescence microscopy as described previously, and plasmid DNA was sequenced to determine proper orientation of the sGFP insert.

## Results

### Stability of pSxFPS1 Reporter Plasmids *in Vitro*

**FIGURE 1:**
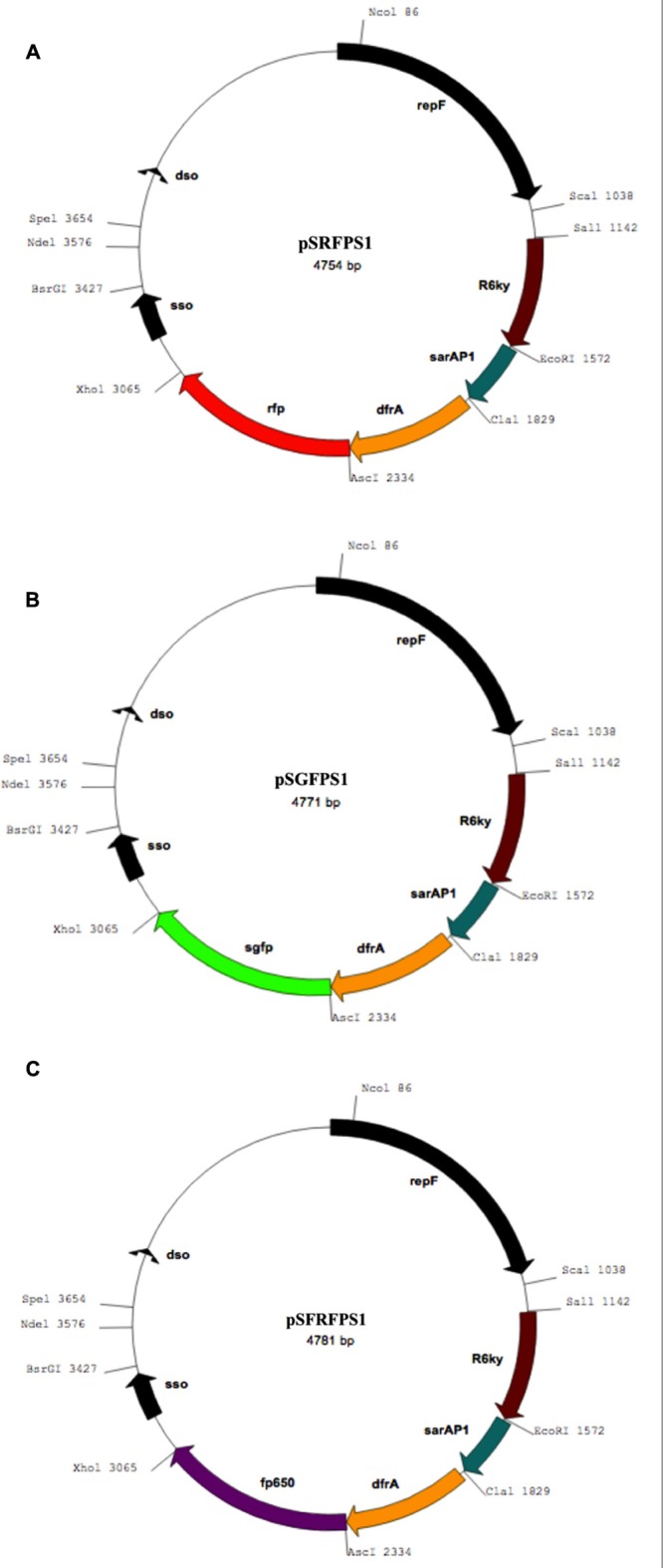
Maps of recombinant plasmids containing genes coding for **(A)** red (pSRFPS1), **(B)** green (pSGFPS1), and **(C)** far-red (pSFRFPS1) fluorescent proteins.

The resulting recombinant reporter plasmids (**Figure 1**) exhibited high stability in both *S. aureus* strains tested. The use of codon-optimized DsRed and eqFP650 proteins ensure desired brightness and detectability for fluorescent imaging techniques and histology work. Non-continuous exponential stability assays demonstrated that the pSxFPS1 reporter constructs remained stable in *S. aureus* SH1000 and RN4220 (**Table 2**). In both strains, the number of colonies on TSA without trimethoprim compared to the number on TSA with trimethoprim remained constant across all three trials. The presence of the plasmid in colonies cultured without antibiotic was confirmed by both fluorescence microscopy and PCR amplification sequencing. All three recombinant strains behaved similarly to pKK30 and were comparably stable, with an average of 99.1% of colonies cultured without trimethoprim capable of growth with the antibiotic across three replicate experients for each recombinant strain (**Table 2**).

### *S. aureus* Reporter Strains Were Cultured from Ovine Tissues

**FIGURE 2:**
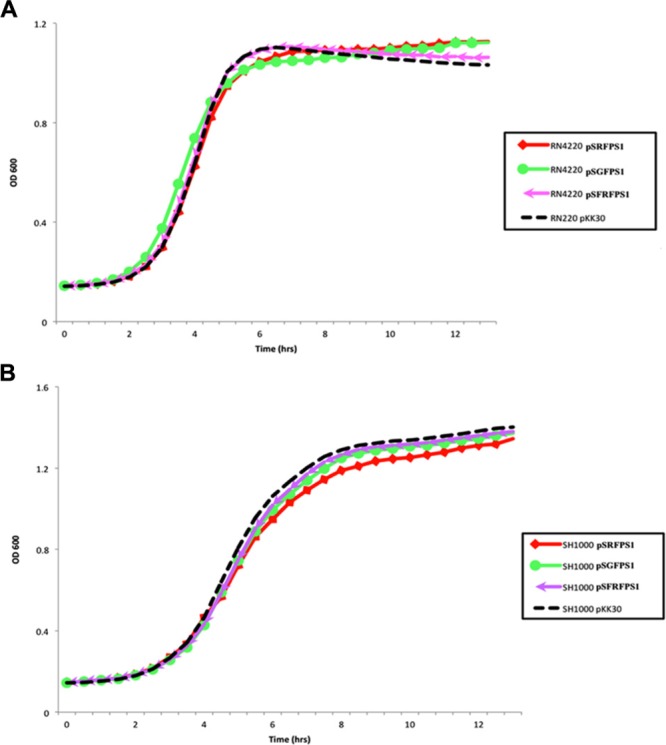
**(A)** Growth of fluorescent strains containing pSGFPS1, pSRFPS1, and pSFRFPS1 in **(A)** RN4220 and **(B)** SH1000 in TSB media without antibiotic. Growth of pKK30 vector without inserts is shown in black.

**FIGURE 3:**
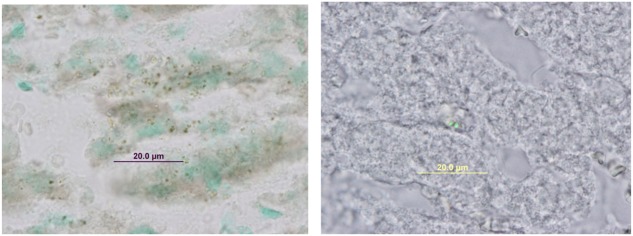
Immunohistochemical staining with anti-GFP in maternal liver infected with *Staphylococcus aureus* SH1000 (pSGFPS1).

**FIGURE 4:**
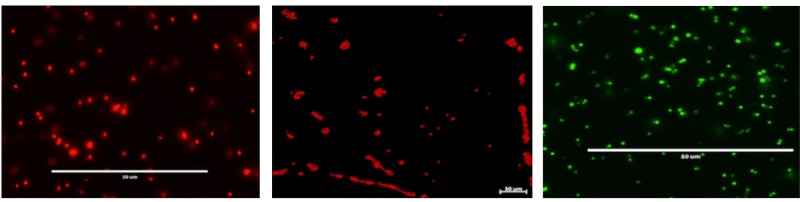
Fluorescent microscopy of cultured *S. aureus* SH1000 strains containing pSRFPS1 **(left)**, pSFRFPS1 **(middle)**, and pSGFPS1 **(right**).

**FIGURE 5:**
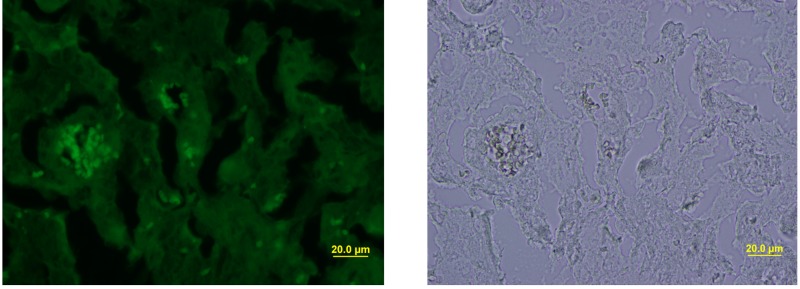
Fluorescence microscopy and bright-field staining of maternal liver infected with *S. aureus* SH1000 (pSGFPS1).

*Staphylococcus aureus* SH1000 (pSGFPS1) was successfully recovered on solid media from maternal tissues following a 6 day incubation period *in vivo*. Bacterial colonies were highly abundant in the placenta, liver, and spleen samples. These cultures also exhibited equivalent brightness when assessed by fluorescent microscopy compared to the inoculum strains (**Figure 2**). Thus, viable, fluorescently labeled *S. aureus* can be cultured from infected animals. In addition to culturing, *S. aureus* (pSGFPS1) cells were detected in placenta and maternal liver (**Figure 3**). GFP-expressing bacteria were detectable in both of these tissues using immunohistochemistry using GFP antibodies (**Figures 4, 5**) and by fluorescence microscopy as described previously. At 10^4^ cells of *S. aureus* SH1000 with a fluorescent plasmid, no sickness or lethality were observed in the ewe or the fetus. However, in subsequent experiments, inoculation with 10^6^ cells caused illness in the ewe while a dose 10^8^ cells was lethal to the ewe and fetus. This result shows that SH1000 maintains virulence with the reporter plasmids *in vivo*.

## Discussion

The three stable reporter plasmids described here allow for the study of the movement and growth of *S. aureus* within animal hosts without the need for any disruption of the bacterial chromosome or the use of any antibiotics to maintain their presence in the bacterial cell. Each reporter plasmids contains a gene coding for a different fluorescent protein. Having these different labels, for example, allows an investigator to inoculate multiple sites simultaneously in order to test routes of infection and reduce the number of animals used in the experiment. The use of *S. aureus* in this study is preferrable due to its efficient colonization and virulence *in vivo*.

Plasmid stability in the absence of selection pressure is another crucial characteristic when the genes of interest are carried on an extrachromosomal replicon. This trait is conferred on these plasmids from the original pKK30 background and the stability phenotype is confirmed here in these new constructs. As a result of plasmid stability during cell division in the absence of selection pressure, an investigator can use a strain carrying any of these plasmids and be confident that the fluorescent cells observed in a tissue accurately reflects the population size in the tissue. Adding antibiotics to maintain plasmid stability can add a tremendous confounder to experiments where an investigator is interested in how a labeled bacterium may interact with the native bacterial populations. Fortunately, these plasmids remove that confounder.

In this study, labeled cells were visualized within sheep tissues 4–6 days after inoculation of the animal by immunocytochemistry and fluorescence microscopy. In addition, culturable *S. aureus* strains containing these plasmids were re-isolated from the sheep tissues 6 days after inoculation. A recent study showed that hypoxic treatment of pregnant ewes resulted in strains of *S. aureus and S. simulans* entering the fetal brain presumably through the placenta (Zarate et al., 2017). Although the genomes of the *S. simulans* strains isolated from the fetal brain and placenta were identical (within the limits of genome sequencing error), inoculation of the pregnant ewe with a *Staphylococcus* strain labeled with one of the plasmids described here would resolve the question of the origin of the strain in the fetal brain.

The National Institute of Allergy and Infectious Diseases of the National Institutes of Health are sufficiently persuaded by the utility of these *S. aureus* – optimized plasmids to make them available through BEI Resources. This will make these plasmids available to the entire community of microbiologists for the foreseeable future.

## Author Contributions

MR did the lab work needed for the project and wrote the first draft of the paper. ZP performed and interpreted the tissue staining done in this work. CW did the animal validation of the bacterial strains used here and edited the paper. KR provided valuable advice on the choice replicons and reporter genes used in this work. ET conceptualized the work, worked on all drafts of the paper, obtained the accession numbers for the plasmids, and worked with BEI Resources to make the plasmids available to the research community.

## Conflict of Interest Statement

The authors declare that the research was conducted in the absence of any commercial or financial relationships that could be construed as a potential conflict of interest.
